# Supplementary material for: Improving probabilistic infectious disease forecasting through coherence
Source: PLoS Comput Biol. 2021 Jan 6;17(1):e1007623. doi: 10.1371/journal.pcbi.1007623 (PMC7837472; doi:10.1371/journal.pcbi.1007623)
Supplement: S1 Appendix — (PDF) [file pcbi.1007623.s001.pdf]

Supporting information

S1 Appendix.

Theorem 1. *Let* $\boldsymbol{X}_{n\times p}$ *be a matrix of full column rank. Assume* $\boldsymbol{y} \in$ *colspace(*$\boldsymbol{X}$*),* $\tilde{\boldsymbol{y}} = \boldsymbol{y} + \boldsymbol{\delta}$ *where* $\boldsymbol{\delta} \in \mathbb{R}^n$*, and* $\hat{\boldsymbol{y}} = \boldsymbol{P}\tilde{\boldsymbol{y}}$*, where* $\boldsymbol{P} = \boldsymbol{X}(\boldsymbol{X}^T\boldsymbol{X})^{-1}\boldsymbol{X}^T$*. Then,* $||\tilde{\boldsymbol{y}} - \boldsymbol{y}||_2 \geq ||\hat{\boldsymbol{y}} - \boldsymbol{y}||_2$.

Proof. Properties for the proof:

Property 1: Let $\boldsymbol{X}$ be an $n \times p$ matrix such that rank($\boldsymbol{X}$) $= p$.

Property 2: Define $\boldsymbol{P} = \boldsymbol{X}(\boldsymbol{X}^T\boldsymbol{X})^{-1}\boldsymbol{X}^T$. Then

- Property 2a: $\boldsymbol{P}$ is idempotent (i.e., $\boldsymbol{P} = \boldsymbol{P}\boldsymbol{P} = \boldsymbol{P}^2$).
- Property 2b: $\boldsymbol{P}$ is symmetric (i.e., $\boldsymbol{P} = \boldsymbol{P}^T$).
- Property 2c: $\boldsymbol{I} - \boldsymbol{P}$ is idempotent.
- Property 2d: $\boldsymbol{I} - \boldsymbol{P}$ is symmetric.

Property 3: If $\boldsymbol{A}$ is a symmetric matrix, then $\boldsymbol{A} = \boldsymbol{Q}\boldsymbol{\Lambda}\boldsymbol{Q}^T$ where $\boldsymbol{Q}$ is a matrix whose columns are equal to the eigenvectors of $\boldsymbol{A}$ and $\boldsymbol{\Lambda}$ is a diagonal matrix whose diagonal elements λ are the eigenvalues of $\boldsymbol{A}$.

Property 4: If $\boldsymbol{A}$ is idempotent, then its eigenvalues are either 0 or 1.

Property 5: Assume $\boldsymbol{y} \in$ colspace($\boldsymbol{X}$).

Property 6: $\tilde{\boldsymbol{y}} = \boldsymbol{y} + \boldsymbol{\delta}$, where $\boldsymbol{\delta} \in \mathbb{R}^n$.

Property 7: $\hat{\boldsymbol{y}} = \boldsymbol{P}\tilde{\boldsymbol{y}}$

The proof proceeds as follows:

$$
\begin{aligned}
||\tilde{\boldsymbol{y}} - \boldsymbol{y}||_2 - ||\hat{\boldsymbol{y}} - \boldsymbol{y}||_2 &= ||\boldsymbol{y} + \boldsymbol{\delta} - \boldsymbol{y}||_2 - ||\boldsymbol{P}\tilde{\boldsymbol{y}} - \boldsymbol{y}||_2 && \text{(Properties 6 and 7)}\\
&= ||\boldsymbol{\delta}||_2 - ||\boldsymbol{P}\boldsymbol{y} + \boldsymbol{P}\boldsymbol{\delta} - \boldsymbol{y}||_2 && \text{(Property 6)}\\
&= ||\boldsymbol{\delta}||_2 - ||\boldsymbol{y} + \boldsymbol{P}\boldsymbol{\delta} - \boldsymbol{y}||_2 && \text{(Property 5)}\\
&= ||\boldsymbol{\delta}||_2 - ||\boldsymbol{P}\boldsymbol{\delta}||_2\\
&= \boldsymbol{\delta}^T\boldsymbol{\delta} - \boldsymbol{\delta}^T\boldsymbol{P}^T\boldsymbol{P}\boldsymbol{\delta}\\
&= \boldsymbol{\delta}^T\boldsymbol{\delta} - \boldsymbol{\delta}^T\boldsymbol{P}\boldsymbol{P}\boldsymbol{\delta} && \text{(Property 2b)}\\
&= \boldsymbol{\delta}^T\boldsymbol{\delta} - \boldsymbol{\delta}^T\boldsymbol{P}\boldsymbol{\delta} && \text{(Property 2a)}\\
&= \boldsymbol{\delta}^T(\boldsymbol{I} - \boldsymbol{P})\boldsymbol{\delta}\\
&= \boldsymbol{\delta}^T\boldsymbol{Q}\boldsymbol{\Lambda}\boldsymbol{Q}^T\boldsymbol{\delta} && \text{(Properties 2d and 3)}\\
&= \boldsymbol{a}^T\boldsymbol{\Lambda}\boldsymbol{a} && \text{(where } \boldsymbol{a} = \boldsymbol{Q}^T\boldsymbol{\delta})\\
&= \sum_{i=1}^{n} \lambda_i a_i^2 && \text{(Property 3)}\\
&\geq 0. && \text{(Properties 4 and 2c)}
\end{aligned}
$$

Thus, $||\tilde{\boldsymbol{y}} - \boldsymbol{y}||_2 - ||\hat{\boldsymbol{y}} - \boldsymbol{y}||_2 \geq 0$ implies $||\tilde{\boldsymbol{y}} - \boldsymbol{y}||_2 \geq ||\hat{\boldsymbol{y}} - \boldsymbol{y}||_2$. □
