# Supplementary material for: Improving probabilistic infectious disease forecasting through coherence
Source: PLoS Comput Biol. 2021 Jan 6;17(1):e1007623. doi: 10.1371/journal.pcbi.1007623 (PMC7837472; doi:10.1371/journal.pcbi.1007623)
Supplement: Attachment — Submitted filename: Response to Reviewers of PLOS Computational Biology.pdf [file pcbi.1007623.s002.pdf]

Response to Reviewers of PLOS Computational Biology
Improving Probabilistic Infectious Disease Forecasting Through Coherence
PCOMPBIOL-D-06567864

Dear Editors,

My coauthors and I thank you for reviewing our work.
Reviewer comments were much appreciated and we took all comments suggested. A detailed list of reviewer comments, our response, and the resulting edits to the manuscript are included below.

We have included the comment point by point and have included our response in blue below.

We appreciate the opportunity to submit a revised version of the manuscript and look forward to hearing from you.

Sincerely,

Graham Gibson

Review # 1

1) The authors mention they use methods inspired from Gamakumara et al. and Clark et al. While this seems to be the first application of forecast coherence in the context of FluSight challenge, the methodological novelty in this paper w.r.t. the problem itself is not clear. Would be good to highlight this through a related work comparison. The authors also state in the Introduction that 'demonstrated benefits of coherence in the point prediction setting do not necessarily translate to the probabilistic realm' ([19] in the submission). [18] seems to talk about coherence for probabilistic forecasting. More discussion is needed.

Response #1: We agree with the authors that the novelty could be made clearer. We expanded on this by clearly explaining the novelty with respect to situations without historical forecast accuracy. Previous approaches required estimating weights on historical data, however, for new teams in the FluSight challenge, emerging epidemic situations, and novel surveillance networks this is not possible. "Previous work has focused on applying probabilistic coherence constraints after estimating regional weights based on historical data. While this is appealing from an optimization perspective, it limits new participants in the FluSight challenge, as well as other applications with limited forecast accuracy data, such as in emerging epidemics and novel surveillance networks." (Page 3)

2) Is the positive correlation assumed by Ordered OLS only between region and national performance, or even among neighboring regions? Figure 7 does show the rationale between national and regional forecasts. In reality, I believe there is a case where the correlation structure is unknown (especially among regions given the underlying strain heterogeneity), and hence ordering forecasts across regions may not be straightforward. This may not be even learn-able from historical data, given the flu seasons have significant spatial variations across years.

Response #2: The positive correlation assumed by ordered OLS induces a correlation structure across all regions. We do this by ordering the samples drawn from all regions before applying the projection matrix. We have added the clarifying sentence, "The correlation induced creates a positive correlation between all the regions and the nation." (Page 11)

3) In discussion, would be good for the authors to comment on preserving short-term and seasonal forecasts (onset/peak) consistency within each method and in the ensemble when altering each of these forecasts for hierarchical consistency. There is also the issue of consistency among 4 short-term forecasts. These are less straightforward to define, but some discussion will help.

Response #3: By applying the projection matrix we enforce "consistency" (coherence) across regions for all targets (1-4 wk ahead). We explicitly removed seasonal targets from the analysis because it is unclear what coherence means for seasonal targets, e.g. if we know the seasonal

peak for all regions, we still do not deterministically know the national peak. We have added the clarifying sentence, " In this analysis, we focus only on short-term (1-4 week ahead) targets. The definition of coherence is less clear on seasonal targets, since knowing the regional season peaks does not inform the national season peak. " (Page 8)

4) While the authors state that a 'bottom up' method ignores national forecasts, it would still serve as a valid baseline for comparison. Are national forecasts always better than regional ones? It's not clear at the outset, given national has higher N but captures the spatiotemporal evolution of flu season only in a coarse sense.

Response #4: We completely agree, and have included bottom-up as a baseline in the evaluation. National forecasts are not always better than regional ones, as demonstrated in Figure 6.

5) The paper seems to use 2010 Census numbers. Does CDC release 'official' weights (i.e., which year's census population estimates to use) for states/regions each influenza seasons? Though the authors report overall correlation, I assume using inexact weights may have impacts on individual forecasts (esp. in single-bin skill).

Response #5: Unfortunately, we do not have access to the true weights, but we include the correlation coefficients … "The correlation between the weighted combination of the regionally reported wILI by the CDC and the nationally reported wILI is >.99" (Page 14).

6) The authors use multi-bin skill for evaluating the forecasts. Does this also translate to improved performance by the single-bin proper scoring rule that CDC is adopting starting 2019-20 season?

Response #6: We again completely agree, since moving forward the CDC requires single-bin scoring. This comment sparked a major restructuring of the manuscript around single-bin scoring. This required some additional methodological development to maintain the same performance gains seen under multi-bin. Major changes included

- Formally defining single-bin scoring. (Page 7)
- Introduction of a weighted projection method, where weights are proportional to normalized population sizes across regions. (Page 10)
- Restructuring the methods evaluated to include all possible permutations (four) of both weighting the forecast distributions and inducing a correlation structure as before. (Page 15)
- Discussing both single and multi bin improvement in forecast skill across all projection methods. (Page 15)
- Highlighting that the choice of scoring rule matters since it changes the optimal performing coherence method. (Page 19)

- Investigate the relationship between the way the projection method changes the variance of the forecast distribution and the relationship between the variance and scoring rule. (Page 19)

7) - References [18] and [20] seem to be duplicates (including page numbers).

Response #7: Corrected.

8) - In Figure (4) use a darker black for true wILI.

Response #8: Corrected.

9) - In Section 2.1, the graphical demonstration of Unordered OLS is cited as Figure 4. Is this correct?

Response #9: Corrected.

10) - In Section 2.3, would be good to explicitly state 'evaluate both approaches on short-term forecasts across epiweeks 44-17'

Response #10: Corrected.

11) - Typo in first paragraph of Section 3. The 'unordered OLS method' saw an improvement in two-thirds...

Response #11: Corrected.

12) Some explanation for the structure of the projection matrix (eqn (5)) will be useful.

Response #12: We included additional explanation of the coherent projection matrix. "We can think of this as a linear regression model with a design matrix X that enforces coherence. Therefore, any projection into the column space of X, must preserve coherence".

13) - What is the y-axis in Figure 5? Is it each epiweek in the three test seasons?

Response #13: The y-axis represents a unique model season combination, which we use as the fundamental unit of analysis when scoring. This has been clarified.

Review # 2

1) "directly computed using state population weighted ILI"
   - this sounds like weighted ILI at the state level rather than the population-weighted average of state ILI

Response #1: We have corrected this for clarity. "At the national level, wILI can be directly computed using a sum of state population weighted ILI ...:"

2) "The CDC estimates ILI as the ratio of patients presenting with a cough and fever equal to or above 100° Fahrenheit over the total number of patients presenting at health care providers [5]"

- It is my understanding that ILI is fever AND (cough OR sore throat). Not sure this is the best reference. Maybe something from ILINet?

Response #2: We agree and have updated the reference,

Thompson, M. G., et al. "Estimates of deaths associated with seasonal influenza-United States, 1976-2007." *Morbidity and Mortality Weekly Report* 59.33 (2010): 1057-1062.

3) - Might be worth providing a little more detail about the FluSight forecasts for the uninitiated reader. Forecast targets, weeks available, etc.

Response #3: We think that the references to fundamental papers in the field serve to introduce the reader to the challenge and accurately describe weeks available and forecast targets, especially since we restrict our attention to the more intuitive week ahead targets.

4) "The CDC reports the wILI data using epidemic weeks, called epiweeks, instead of calendar weeks [21]."
   - I wasn't able to find this source. add url or use something from MMWR

Response #4: Corrected to https://ibis.health.state.nm.us/resource/MMWRWeekCalendar.html.

5) "We score the probabilistic forecasts using multi-bin skill, rather than multi-bin log score as used in the FluSight challenge......"
   - Someone unfamiliar with the challenge may have trouble understanding this paragraph. Maybe start by introducing the set of bins Z, by which FluSight probabilistic forecasts are submitted. Then summarize skill and log score before continuing with the paragraph.

Response #5: We agree and have rearranged the paragraph to more clearly introduce the scoring.

6) "Previous approaches have factored...[18]"
   - Text implies there should be more than one reference
   - Figure 4: vertical line for true wILI is difficult to distinguish from grid.

Response #6: We have corrected the sentence. “Taieb et. al factored $f(\tilde{\bm{y}})$ into a bottom-up density, where..” and corrected the plot

7) - Algorithm 2 might be more clear if it explicitly includes a step for sorting along the i index. Otherwise the reader must catch a minor change of notation to see the difference from Algorithm 1.
   - Figure 5: axis tick marks are too small. Caption has typos
   - Figure 6: specify box and whisker extents/limits

Response #7: We agree, and have included an additional sort step.

8) "In fact, the breakdown obscures any improvement at all, a consequence of using forecast skill as the primary metric, which is a geometric mean of log score."
   - Log score has not been defined. It is not self-evident that this comment is true.
   - Figure 7: what are the error units? wILI? weeks? both?

Response #8: This sentence is no longer relevant, since moving to single-bin we see a significant increase in forecast skill when broken down by region as well.
