# Supplementary material for: Improving probabilistic infectious disease forecasting through coherence
Source: PLoS Comput Biol. 2021 Jan 6;17(1):e1007623. doi: 10.1371/journal.pcbi.1007623 (PMC7837472; doi:10.1371/journal.pcbi.1007623)
Supplement: Attachment — Submitted filename: Response 2 to Reviewers of PLOS Computational Biology.pdf [file pcbi.1007623.s003.pdf]

Response to Reviewers of PLOS Computational Biology
Improving Probabilistic Infectious Disease Forecasting Through Coherence
PCOMPBIOL-D-06567864

Dear Editors,

My coauthors and I thank you for reviewing our work.
Reviewer comments were much appreciated and we took all comments suggested. A detailed list of reviewer comments, our response, and the resulting edits to the manuscript are included below.

We have included the comment point by point and have included our response in blue below.

We appreciate the opportunity to submit a revised version of the manuscript and look forward to hearing from you.

Sincerely,

Graham Gibson

Reviewer # 1

1) The acronym 'WOLS' appears in captions prior to being defined.

Response #1: We have defined WOLS in the caption itself on Figure 4 (page 11) and Algorithm 1 (page 12).

2) Table 2 caption and text reference does not make clear that "percent increase" refers to "percent of forecasts improved". Globally, consider replacing statements like 'XX% improvement' with 'XX% of forecasts improved'

Response #2: We agree with the reviewer and have clarified the meaning in Table 2 (Page 15) to explicitly refer to “percent of forecasts improved”. We also changed the language of ‘XX% improvement’ with ‘XX% of forecasts improved’ in the discussion on page 18 and 20.

3) - Discussion, first bullet: "However, the some models..." should probably be "However, some models..."

Response #3: We agree with the reviewer and have made the suggested change.

4) - Discussion, fifth bullet: "Under the 17 models" might sound better "For the 17 models"

Response #4: We agree with the reviewer and have made the suggested change.
